# Altered Gene Expression in Blood and Sputum in COPD Frequent Exacerbators in the ECLIPSE Cohort

**DOI:** 10.1371/journal.pone.0107381

**Published:** 2014-09-29

**Authors:** Dave Singh, Steven M. Fox, Ruth Tal-Singer, Stewart Bates, John H. Riley, Bartolome Celli

**Affiliations:** 1 University of Manchester, Medicines Evaluation Unit, Manchester, United Kingdom; 2 GlaxoSmithKline, Medicines Research Centre, Stevenage, United Kingdom; 3 GlaxoSmithKline, King of Prussia, Pennsylvania, United States of America; 4 GlaxoSmithKline, Stockley Park, Uxbridge, United Kingdom; 5 Brigham and Women's Hospital and Harvard Medical School, Boston, Massachusetts, United States of America; Helmholtz Zentrum München/Ludwig-Maximilians-University Munich, Germany

## Abstract

Patients with chronic obstructive pulmonary disease (COPD) who are defined as frequent exacerbators suffer with 2 or more exacerbations every year. The molecular mechanisms responsible for this phenotype are poorly understood. We investigated gene expression profile patterns associated with frequent exacerbations in sputum and blood cells in a well-characterised cohort. Samples from subjects from the ECLIPSE COPD cohort were used; sputum and blood samples from 138 subjects were used for microarray gene expression analysis, while blood samples from 438 subjects were used for polymerase chain reaction (PCR) testing. Using microarray, 150 genes were differentially expressed in blood (>±1.5 fold change, p≤0.01) between frequent compared to non-exacerbators. In sputum cells, only 6 genes were differentially expressed. The differentially regulated genes in blood included downregulation of those involved in lymphocyte signalling and upregulation of pro-apoptotic signalling genes. Multivariate analysis of the microarray data followed by confirmatory PCR analysis identified 3 genes that predicted frequent exacerbations; B3GNT, LAF4 and ARHGEF10. The sensitivity and specificity of these 3 genes to predict the frequent exacerbator phenotype was 88% and 33% respectively. There are alterations in systemic immune function associated with frequent exacerbations; down-regulation of lymphocyte function and a shift towards pro-apoptosis mechanisms are apparent in patients with frequent exacerbations.

## Introduction

Exacerbations of COPD are defined as an acute worsening of symptoms beyond the daily variability seen in patients with COPD and are associated with increased airway and systemic inflammation [1]. Exacerbations are commonly triggered by viruses or bacteria, although other environmental trigger factors such as air pollution are recognised [1], [2]. The ECLIPSE study has recently identified a frequent exacerbation phenotype present across all GOLD airflow limitation stages, characterized by developing at least 2 exacerbations every year over a 3 year follow up [3]. In the same study there were subjects at all GOLD stages who did not exacerbate at all over three years. Patients with more frequent exacerbations are known to have worse quality of life and increased mortality [4], [5].

The cellular and molecular mechanisms responsible for the increased susceptibility to exacerbations in the frequent exacerbation phenotype are poorly understood. If the cascade of inflammatory events that result in the clinical development of an exacerbation episode is centred in the lungs, it is likely that there are differences in the airway cells of patients with the frequent exacerbation phenotype compared with those that do not have exacerbations. However, if the cascade represents a generalized systemic response to pathogens or other trigger factors, it is likely that there will be differences that could be detected in immune cells in the systemic circulation.

We hypothesized that there are differences in the gene expression profile in the blood and airway cells of frequent exacerbators compared with non-exacerbators. To test this hypothesis we studied well characterized COPD subjects in the ECLIPSE cohort. We investigated the gene expression profile pattern associated with the frequent exacerbation phenotype in sputum and blood cells.

## Methods

### Subjects

ECLIPSE is a 3-year multicentre longitudinal study to identify novel endpoints in COPD; the methodology has been previously described [6]. Sputum induction was performed and blood samples obtained in a subset of 148 COPD ex-smokers at 14 sites at the start of the study. Samples of sufficient quality for gene array analysis were obtained from 138 of these subjects. These subjects were subsequently followed up for 3 years, and the number of exacerbations was quantified. Blood samples obtained from a different group of 215 COPD patients participating in ECLIPSE were used for PCR analysis.

### Ethics statement

ECLIPSE was ethically approved by the local ethics committee at each participating centre; Clinicaltrials.gov identifier NCT00292552; GSK Study Identifier SCO104960. All participants provided written informed consent.

### Sputum induction and processing

The methods for sputum induction and processing have been previously described [7] and are included in the supporting information (File S1).

### Whole blood collection

Using standard venipuncture techniques, 2.5 mls of blood was drawn into each of two PAXGene blood collection tubes. The isolation of RNA from these samples is described in the supporting information (File S1).

### Microarray processing

The performance of microarrays is described in the supporting information (File S1).

### Real time PCR

RNA was isolated and processed by Aros Applied Biotechnology (Denmark) as described in the supporting information (File S1).

### Statistical analysis

Patients with frequent exacerbations were defined as those who had experienced two or more exacerbations requiring oral corticosteroids and/or antibiotics or were hospitalised within a year, for each of the 3 years of the study as previously defined in the ECIPSE study [3]. This group was compared to patients with no exacerbations during this time period. Univariate analysis used a p value of <0.01 to define significant differences between groups, and gene expression fold change (FC) levels as indicated in the text. Individual genes were mapped to Genego pathways (GeneGo, St. Joseph, MI, http://www.genego.com/metacore.php), with p≤0.01 and FDR<0.05 used to identify significant pathways. The gene array data is accessible at geo@ncbi.nlm.nih.gov (GEO ID GSE4837 and GSE22148).

**Figure 1:**
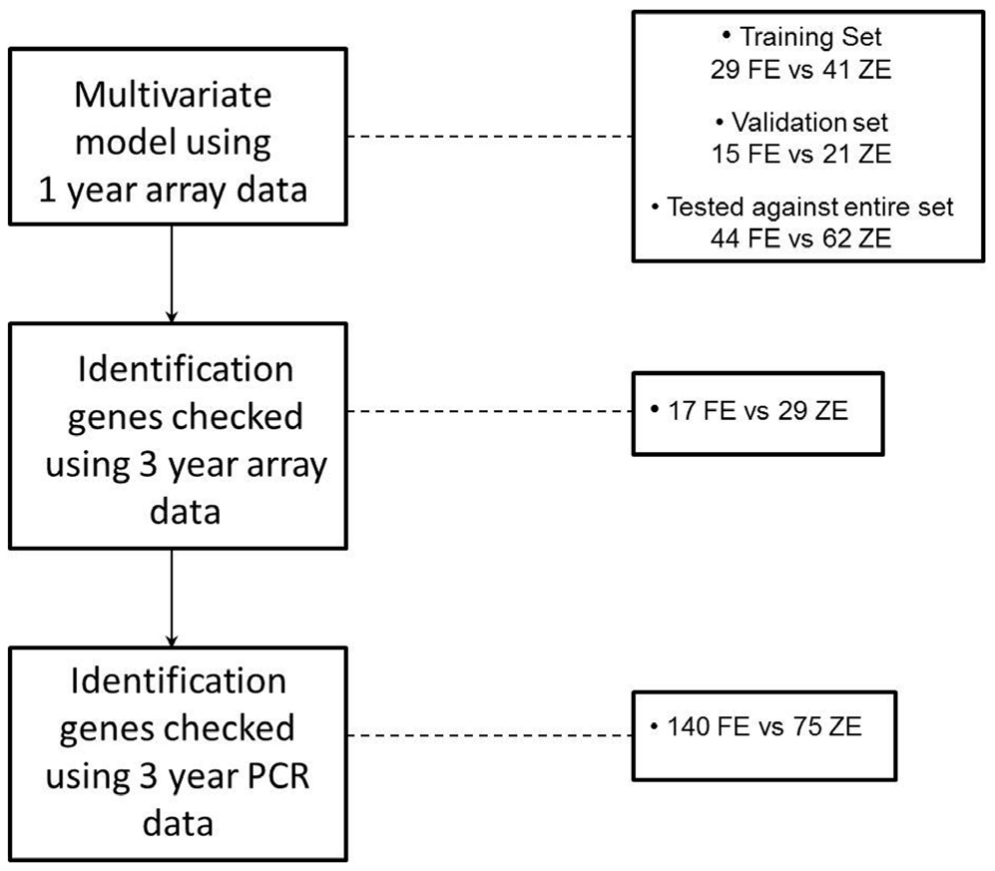
Identification of a set of genes in blood associated with the frequent exacerbator phenotype; multivariate analysis using micro-array data 1 year follow up data (n = 106), followed by univariate analysis of 3 year follow up data (n = 46) and univariate analysis of a different population (n = 215) by PCR. FE =  frequent exacerbators; ZE  =  zero exacerbators.

A linear model analysis of variance was also used to identify a set of genes associated with the frequent exacerbator phenotype; The analysis was adjusted for age, gender and batch by including these variables as terms in the model. Previous subject reported exacerbation history was also used as one of the variables in the model. The history of exacerbations after one year was used to create the model, as there were more subjects with either frequent or zero exacerbations after one year (44 versus 62 respectively) compared to three years (17 vs 29 respectively). The subjects were randomly split into a training set and a validating set using the data at year 1 as shown in Fig. 1. The training set included 29 subjects with frequent exacerbations and 41 with zero exacerbations and for the validating set these numbers were 15 and 21 respectively. 100 training sets were used to create 100 predictive models. Stepwise logistic regression was used to select the most significant predictors; The model structure was (ln[p/(1-p)] =  *α*+*β_1_*(predictor1) + *β_2_*(predictor2)…. + e), p is the probability that the subject will have 2 or more exacerbations and p/(1-p) is the “odds ratio” and ln[p/(1-p)] is the log odds ratio, or “logit”. Each model was checked against the 1000 validating sets to determine its prediction accuracy. One of the best performing models was then checked against the whole sample set of 44 frequent exacerbators compared to 62 zero exacerbators.

Real time PCR data were similarly analyzed using a linear model analysis of variance. A covariate was also included to account for any change in expression due to the RNA loading of the samples. This covariate was represented by the scores from the first principal component obtained from a PCA analysis of the two housekeeper genes.

## Results

### Microarray data

The demographic and clinical characteristics at baseline for the 138 COPD patients whose sputum and blood samples were used for gene arrays are shown in the supporting information (Table S1 in File S1). After 3 years of follow up, there were 117 patients who completed the study with a documented exacerbation history; the demographic details of these subjects are shown in Table 1; 17 patients were frequent exacerbators and 29 patients had zero exacerbations.

**Table 1:** Demographics of the COPD subjects for microarray analysis.

	All	Zero	Intermediate	Frequent
N	117	29	71	17
GOLD Stage II (%)	51	55	55	29
GOLD Stage III (%)	41	38	39	53
GOLD Stage IV (%)	8	7	6	18
Male (%)	66	69	65	65
Mean age	64.9	63.2	65.7	64.8
Mean pack years	46.8	52.0	45.3	43.7
Percent Predicted FEV1 (%)	50.3	51.8	51.7	42.3
LABA (%)	78.6	58.6	83.1	94.1
Inhaled corticosteroid (%)	75.2	55.2	80.3	88.2
Blood WBC	7.5	7.5	7.3	8.2
Blood neutrophil count	4.9	4.8	4.8	5.5
Blood eosinophil count	0.24	0.21	0.24	0.27
Blood leukocyte count	1.87	2.05	1.82	1.80
Blood monocyte count	0.46	0.43	0.44	0.59

Exacerbations were defined over a 3 year follow up; frequent denotes 2 or more exacerbations each year, zero denotes no exacerbations in any year, and intermediate denotes patients who did not fit the frequent or zero exacerbation phenotype. Blood counts are mean values (X10^9^ cells/L).

There were no differences between groups in the blood counts (p>0.05 for all comparisons between groups). A total of 150 genes (166 probesets) were differentially expressed in blood at the level of>±1.5 FC (p≤0.01) between frequent compared to zero exacerbators. In contrast, in the sputum cells, there were only 6 genes (9 probesets) differentially expressed between these groups. The most highly regulated genes in blood and sputum cells are shown in Table 2, with a full list of genes differentially expressed>±1.5 FC in the supporting information (Table S2 in 1).

**Table 2:** The 10 most highly regulated genes in sputum and blood from microarray analysis; a positive fold change  =  increase in frequent exacerbators compared to zero exacerbators, a negative fold change  =  decrease in frequent exacerbators compared to zero exacerbators.

Gene Name	Affy. ID	Fold change	p value	Sample
LOC284723	232245_at	1.71	0.009	Sputum
ANKRD28	213035_at	1.71	0.008	Sputum
LOC284723	1559977_a_at	1.65	0.009	Sputum
LDHAL6B	210712_at	1.55	0.001	Sputum
MIA	206560_s_at	1.53	0.008	Sputum
OASL	205660_at	−1.54	0.009	Sputum
RHCE	215819_s_at	2.25	0.010	Blood
CCNA1	205899_at	2.23	0.009	Blood
ITGB2	236988_x_at	2.21	0.008	Blood
COL4A3	222073_at	−2.05	0.001	Blood
FCRL1	235982_at	−2.06	<0.001	Blood
FCRL2	221239_s_at	−2.06	<0.001	Blood
CD200	209583_s_at	−2.09	0.002	Blood
HLA-DQB1	212999_x_at	−2.22	0.009	Blood
TCL6	219840_s_at	−2.59	<0.001	Blood
HLA-DQA1	236203_at	−3.27	0.003	Blood

Affy. ID  =  affymatrix identification number. There was no overlap between sputum and blood for highly expressed genes.

**Figure 2:**
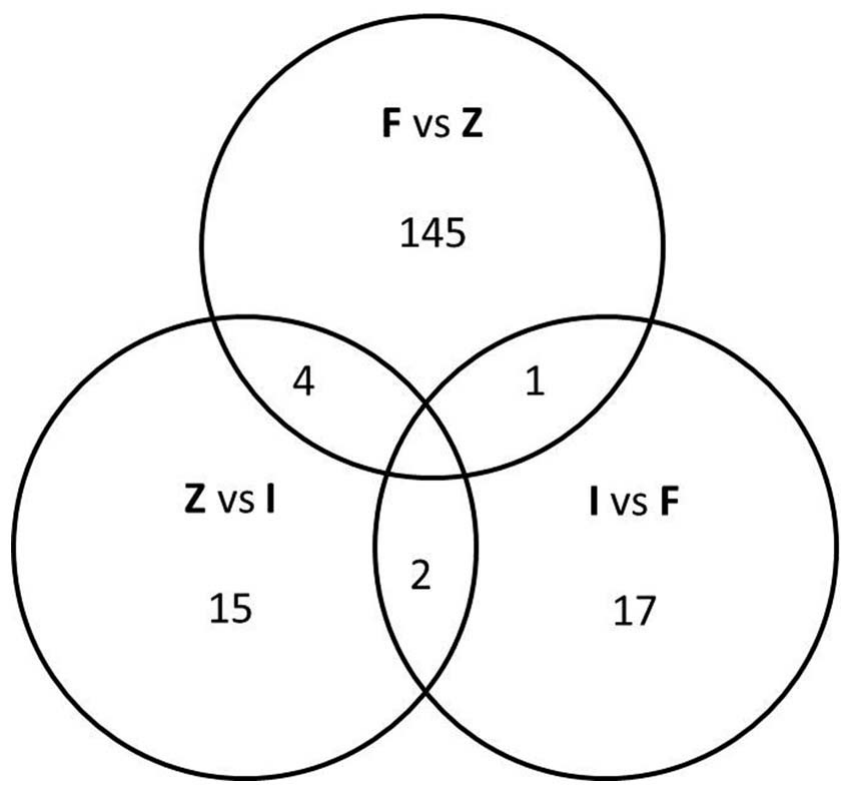
Venn diagram showing the number of differentially regulated genes in blood (fold change +/−1.5 and p<0.01) between frequent exacerbators (F), the intermediate group (I), and zero exacerbators (Z); for example, F vs I denotes number of differentially regulated genes between frequent exacerbators and the intermediate group.

The intermediate group of patients (n = 71) who had exacerbations but did not meet the criteria for inclusion in the frequent exacerbation group had only 21 genes differentially regulated in blood compared to zero exacerbators; there was a greater separation of gene expression in the frequent exacerbation group compared to zero exacerbators (150 genes). The genes differentially expressed in blood from the intermediate group compared to zero exacerbators (21 genes) and compared to frequent exacerbators (20 genes) are shown in the supporting information (Tables S3 and S4 in File S1). Fig. 2 shows that from the 21 genes differentially expressed in intermediate group compared to zero exacerbators, 4 were also differentially expressed in the frequent exacerbators compared to zero exacerbators (listed in supporting information).

### Pathway analysis

As there were relatively fewer gene expression changes observed in the sputum cells of frequent exacerbators compared to zero exacerbators, the rest of the analysis focused on whole blood gene expression. Genego pathway analysis was performed on 811 genes with FC>±1.2 between frequent compared to zero exacerbators. There were 35 significant pathways (p≤0.01, FDR<0.05) where at least 4 genes were regulated, including 14 apoptosis pathways, 8 immune response pathways and 5 cell development pathways; these pathways are shown in the supporting information (Table S5 in File S1). The 5 most highly regulated pathways are shown in Table 3, and included 2 apoptosis signalling pathways (ceramide and lymphotoxin beta receptor [LTBR] signalling) and 3 lymphocyte signalling pathways (inducible T-cell costimulator [ICOS] and CD28 signalling in T cells, and B cell receptor signalling).

**Table 3:** GeneGo pathway mapping of the microarray data for the comparison of zero vs frequent exacerbators; the 5 most highly regulated pathways are shown.

Significance ranking	Pathway Name from GENEGO	Genes	P value	Affy. ID	Fold Change	P value	Gene Name
1	Apoptosis and survival_Ceramides signaling pathway 3072	8/46	0.0000010	222880_at	−1.37	0.009	AKT3
				209364_at	1.34	0.005	BAD
				211833_s_at	1.21	0.002	BAX
				203685_at	−1.29	<0.001	BCL2
				200766_at	1.25	0.004	CTSD
				229415_at	−1.48	<0.001	CYCS
				226046_at	−1.40	0.007	MAPK8
				235252_at	1.24	0.004	KSR1
2	Immune response_ICOS pathway in T-helper cell 619	8/46	0.0000045	222880_at	−1.37	0.009	AKT3
				209364_at	1.34	0.005	BAD
				206545_at	−1.48	0.002	CD28
				1555766_a_at	1.25	0.004	GNG2
				226878_at	−1.40	<0.001	HLA-DOA
				236203_at	−3.27	0.003	HLA-DQA1
				212999_x_at	−2.22	0.009	HLA-DQB1
				210439_at	−1.50	0.002	ICOS
				228976_at	−1.47	0.007	ICOSLG
				202490_at	−1.37	<0.001	IKBKB
				216944_s_at	−1.37	0.005	ITPR1
				228442_at	−1.33	0.006	NFATC2
				204484_at	−1.30	<0.001	PIK3CA
3	Immune response_CD28 signaling	8/54	0.000016	222880_at	−1.37	0.009	AKT3
				209364_at	1.34	0.006	BAD
				206545_at	−1.48	0.002	CD28
				216944_s_at	−1.37	0.004	ITPR1
				226046_at	−1.40	0.007	MAPK8
				228442_at	−1.33	0.006	NFATC2
				209615_s_at	1.29	0.003	PAK1
				204484_at	−1.30	<0.001	PIK3CA
4	Immune response_BCR pathway 655	8/54	0.000016	222880_at	1.37	0.009	AKT3
				209364_at	1.34	0.005	BAD
				203685_at	−1.29	<0.001	BCL2L1
				207655_s_at	−1.49	<0.001	BLNK
				206398_s_at	−1.67	0.002	CD19
				204581_at	−1.62	0.001	CD22
				205544_s_at	−1.79	<0.001	CR2
				212827_at	−1.42	0.002	IGHM
				216944_s_at	−1.37	0.005	ITPR1
				228442_at	−1.33	0.006	NFATC2
				204484_at	−1.30	<0.001	PIK3CA
5	Apoptosis and survival_Lymphotoxin-beta receptor signaling 740	7/41	0.000021	211554_s_at	1.28	<0.001	APAF1
				211833_s_at	1.21	0.002	BAX
				229415_at	−1.48	<0.001	CYCS
				203005_at	1.25	0.008	LTBR
				226046_at	−1.40	0.007	MAPK8
				207907_at	1.29	0.007	TNFSF14
				204352_at	−1.32	0.002	TRAF5

The number of genes regulated within each pathway are shown.

Within the apoptosis pathways, the well recognised pro-apoptotic genes BAD, BAX and LTBR had increased expression, while the anti-apoptotic gene BCL2 had decreased expression, indicating a shift towards pro-apoptotic signalling. Within the lymphocyte signalling pathways, there was decreased expression of genes involved in T cell activation such as the co-stimulatory molecules CD28 and ICOS, the transcription factors NFATC2 and AKT3 and HLA genes that encode MHC proteins responsible for antigen presentation to T cells. B cell activation genes also showed decreased expression, including CD19, complement receptor 2 (CR2) and B-cell linker protein (BLNK), although the B cell regulatory protein CD22 had increased expression. There was also decreased expression of other proteins involved in B cell function such as the FC like receptor (FCLR) family members FCLR1, 2 and 5.

### Multivariate analysis

Multivariate analysis of the gene array data was performed to identify a set of genes most closely associated with the frequent exacerbator phenotype. The 17 and 29 patients followed over three years was an insufficient sample size to create a training and validation set. As described in the methods and Fig. 1, the clinical history of exacerbations after 1 year was used to create the training and validation set, as there were more subjects who experienced no exacerbations and frequent exacerbations in this time period (62 and 44 respectively). Multivariate analysis was performed using 368 genes with FC>±1.2, p≤0.01 between these groups. The best performing model included these 6 genes; SYT6, ARHGEF10, PHPT1, MGC31963, LAF4 and B3GNT (Table 4).

**Table 4:** Gene expression changes in the 6 gene panel identified by microarray analysis using the phenotype data after 1 year.

	Microarray; 1 year phenotype		Microarray; 3 year phenotype		PCR; 3 year phenotype	
	Fold Change	P Value	Fold Change	P Value	Fold Change	P Value
B3GNT1	−1.4	<0.0001	−1.5	<0.0001	−1.2	<0.0001
SYT6	1.4	<0.0001	1	0.881	1	0.693
LAF4	−1.5	<0.0001	−1.7	0.0007	−1.4	<0.0001
ARHGEF10	−1.4	0.013	−1.3	0.0439	−1.4	<0.0001
MGC31963	1.4	0.0001	1.6	0.00046	1	0.5601
PHPT1	1.2	0.003	1.4	0.007	−1.3	<0.0001
FCL5	−1.5	0.01	−1.83	0.003	−1.16	0.131
PLCL2	−1.3	0.002	−1.43	0.006	−1.65	0.0008

The gene expression changes using the phenotype data after 3 years from the microarray population and PCR population are shown. Positive fold change  =  increase in frequent exacerbators compared to zero exacerbators, negative fold change  =  decrease in frequent exacerbators compared to zero exacerbators.

The expression of these 6 genes between frequent and zero exacerbators using 3 year follow up clinical history is shown in Table 4; ARHGEF10, PHPT1, MGC31963, LAF4 and B3GNT were significantly differentially expressed between groups, but not SYT6.

### PCR analysis

PCR analysis of the 5 genes shown by microarray to be differentially expressed in frequent and zero exacerbators after 3 years (ARHGEF10, PHPT1, MGC31963, LAF4 and B3GNT) was performed in a different group of 215 subjects (see Table 5 for demographic details), including 75 with zero exacerbations and 140 with frequent exacerbations in the 3 year follow up period. PLCL2 and FCL5, which were also differentially expressed on microarray, and SYT6 were also analysed by PCR.

**Table 5:** Demographics of the COPD subjects for PCR analysis.

	None	Frequent
N	75	140
Stage II (%)	44	30
Stage III (%)	31	42
Stage IV (%)	25	28
Male (%)	65	56
Mean age	63	63
Mean pack Years	56	48
Percent predicted FEV1 (%)	48	42
Current smokers (%)	38.6	37.6
LABA (%)	45	89
Inhaled corticosteroid (%)	51	94
Blood WBC	7.7	8.2
Blood neutrophil count	5	5.4
Blood eosinophil count	0.24	0.26
Blood leukocyte count	0.44	0.52
Blood monocyte count	2.02	1.95

Exacerbations were defined over a 3 year follow up; frequent denotes 2 or more exacerbations each year, zero denotes no exacerbations in any year, and intermediate denotes patients who did not fit the frequent or zero exacerbation phenotype. Blood counts are mean values (X10^9^ cells/L).

B3GNT1, LAF4, ARHGEF10 and PLCL2 expression were significantly different between frequent and zero exacerbators (see Table 4). SYT6, MGC31963 and FCL5 did not achieve statistical significance, while PHPT1 had a complete reversal of signal. B3GNT, LAF4 and ARHGEF10 were retested as a model in this population; these 3 genes predicted the frequent exacerbation phenotype with sensitivity and specificity of 88% and 33% respectively, with the values improving to 91% and 81% respectively when the exacerbation history in the previous year was considered. Modelling previous exacerbation history alone gave sensitivity and specificity of 90% and 83% respectively.

## Discussion

This study has demonstrated differences in the gene expression profile of COPD patients with frequent exacerbations compared to those with no exacerbations over a 3 year period in ECLIPSE. There were 150 genes differentially expressed in the circulating cells of the two groups, with only 6 differentially regulated genes in sputum cells. This suggests alterations in systemic immune function that contribute to the frequent exacerbation phenotype rather than a specific pulmonary immune defect.

Our results suggest that future investigations into the pathophysiology of COPD exacerbations should focus on changes that occur in the systemic immune system. We have not proved mechanistically that the changes observed here are the cause or consequence of frequent exacerbations, but nevertheless provide a starting point for further investigations into the gene expression differences observed. Microarray gene expression profiling is often “hypothesis generating”, and our results have generated a number of hypotheses regarding potentially dysregulated immunological pathways in COPD frequent exacerbators. We have provided a degree of validation of our findings by performing PCR analysis in a different group of COPD patients. The microarray gene expression findings reported here serve as a basis for future investigations of potential mechanisms involved in COPD exacerbations.

A subset of the microarray gene expression findings were investigated in a different, and larger, group of patients by PCR. Four out of seven microarray genes that showed significant differences in blood using the 3 year follow up data were also significantly different in the PCR analysis in a separate population. Microarray gene expression analysis often produces false positive results due to multiple testing, so it is desirable to validate the findings using different techniques and/or a different sampling population. A major strength of this study is the use of two cohorts for replication. The PCR analysis indicates that many of the micro-array results are not false positive findings. Our pathway analysis describes a large number of genes involved in lymphocyte signalling and apoptosis pathways, and the presence of some false positive data would not change these overall findings.

Our findings are not due to sampling or methodological issues; using the same sputum samples from the subjects in this study, we have recently shown a large set of genes associated with disease severity defined by FEV_1_ or the degree of emphysema at the FC level of 2 [7]; 277 genes were differentially regulated in severe compared to moderate COPD, and 198 genes were differentially regulated according to the degree of emphysema respectively. In stark contrast, there were no induced sputum genes regulated at this FC level in frequent exacerbators compared to zero exacerbators. Furthermore, the gene expression differences in whole blood were not due to differences in the composition of the leukocytes, as the differential white cell counts were similar in patients with frequent exacerbations compared to those with zero exacerbations.

Exacerbations are often caused by respiratory pathogens, such as viruses and bacteria [1], [2]. It is thought that an exaggerated and/or prolonged immune response to pathogens in susceptible COPD patients leads to an acute exacerbation. One might reasonably assume that patients with frequent exacerbations would have changes within the pulmonary immune system that pre-dispose to an exaggerated immune response after exposure to a respiratory pathogen. Alternatively, the abnormality could be systemic in nature. Our results suggest dysregulation of systemic immune function in COPD frequent exacerbators rather than dysfunction limited to the lungs.

### Systemic immunological response

There was decreased expression of the T-cell receptor co-stimulatory molecules CD28 and ICOS, and GeneGo analysis identified multiple changes within these signalling pathways. Co-stimulatory signals through CD28 and ICOS enhance T cell activation following T-cell receptor stimulation [8]. The expression of HLA genes that encode MHC class II was also reduced, suggesting a diminished capacity for antigen presentation to T cells [9]. Reduced antigen presentation coupled with reduced co-stimulation indicates a decreased adaptive immune response in frequent exacerbators. Furthermore, there was also decreased expression of T cell transcription factors such as NFATC2 in frequent exacerbators; this transcription factor is involved in T cell cytokine production [8], [10], and reduced gene expression levels may be a downstream consequence of decreased T cell receptor signalling. There were other T cell specific genes that were highly regulated (Table 2), including decreased expression of> 2 FC for CD200 which is known to suppress inflammatory lymphocyte responses [11] and TCL-6 [12] which is expressed in T cell leukaemia cells. The altered expression of so many genes involved in T cell signalling strongly suggests that altered T cell function plays a mechanistic role in the frequent exacerbator phenotype.

There was decreased expression of B cell activation genes including the co-receptors CD19 and CR2, and the downstream signalling molecule BLNK [13], [14]. CR2, also known as CD21, binds to complement attached to immune complexes; CR2 expression is decreased in auto-immune diseases [15], which may represent physiological down-regulation rather than the primary cause of auto-immunity. In the context of COPD exacerbations, it is also possible that reduced B cell signalling is a primary physiological mechanism that causes susceptibility to exacerbations, or a physiological response to repeated or prolonged infection. The expression of the B cell regulatory protein CD22 [16] was increased in frequent exacerbators, again compatible with negative regulation of B cell function.

There is evidence of impaired T-cell receptor signalling in autoimmune diseases [17], [18]. Similarly, it has recently been shown that the expression of T-cell receptor signalling components is reduced in pulmonary CD8 cells from COPD patients [19]. Our findings now suggest that peripheral blood T and B cells are in a state of decreased activation in COPD patients with frequent exacerbations. This may reduce their ability to act effectively during infections, thus leading to an increased susceptibility to exacerbations.

Pulmonary lymphoid follicles numbers are increased in COPD patients [20]; These organised structures facilitate antigen presentation, cytokine secretion and antibody production by B cells. The exact nature of the antigen presentation is unknown, and may be self-antigens or pathogen derived antigens. It would be interesting to know if there is also reduced activation of cells within lymphoid follicles of COPD exacerbators.

### Cell death and tissue repair

Apoptosis is the process of programmed cell death that occurs as part of normal tissue homeostasis. We observed a shift towards pro-apoptotic mechanisms in peripheral blood samples of frequent exacerbators, as the expression of the pro-apoptotic genes BAD [21], BAX [21] and LTBR [22] was increased, while anti-apoptotic BCL2 [21] expression was decreased. This suggests that immune cells in the peripheral circulation of frequent exacerbators have an increased rate of apoptosis. There is evidence of increased apoptosis in the lungs of COPD patients [23], which may be related to increased levels of oxidative stress. A balance of increased tissue apoptosis with reduced clearance by immune cells can lead to increased tissue inflammation. Immune cells cannot participate in the normal immune response while undergoing programmed cell death; this may be an important mechanism contributing to a reduced immune response against pathogens in frequent exacerbators.

### Other pathway genes

Table 2 shows some relatively highly regulated genes that may be of biological relevance in COPD. For example, CCNA1 encodes the protein cyclin A1 which is involved in cell cycle processes [24] and was increased in frequent exacerbators. Increased CCNA1 may indicate increased cell cycle in blood cells capable of cell division, such as lymphocytes. There was also increased ITGB2 expression; this gene encodes CD18 which is involved in leukocyte adhesion [25].

The 3 gene panel confirmed by PCR were B3GNT1 which encodes beta-1,3-N-acetylglucosaminyltransferase enzyme involved in poly-N-acetyllactosamine synthesis [26], LAF4 which is a transcription factor involved in lymphoid development [27], and ARHGEF10 which is a rho GTPase involved in cell signalling events [28]. The expression of these genes was reduced in frequent exacerbators. Reduced LAF4 expression is compatible with our other findings of decreased lymphocyte signalling in frequent exacerbators. It is unclear if our findings regarding B3GNT1 and ARHGEF10 are indicative of mechanisms involved in frequent exacerbations, or biomarkers of patients who have such events. Further studies of the function of these genes, and the other significantly regulated genes reported here, in COPD patients would shed light on their potential biological roles. It would also be of value to study the protein expression levels of these genes to provide further validation of our findings.

### Comparison with previous studies

There are other studies that have investigated gene expression profiles in COPD peripheral blood using isolated cells [29], [30]. The novelty of our work is that we have investigated gene expression associated with exacerbation history; previous studies have not addressed this question. The interpretation of these previous studies is often restricted by relatively small sample sizes. However, a recent large study (n = 211) investigated gene expression associated with the presence and severity of COPD [30]. Interestingly, there were some findings in common with our study; T cell receptor signalling was found to be altered, with PLCL2, which is involved in signal transduction processes, being one of the differentially regulated genes [31].

### Multivariate analysis

Multivariate modelling identified 3 genes in blood samples that could be used with high sensitivity (91%) to predict the frequent exacerbator phenotype. The sensitivity of this 3 gene panel was as good as the clinical history (90%). A previous history of exacerbations is a good predictor of future exacerbations [5], and our results confirm that clinical history is reliable and sensitive in this regard. We do not suggest this gene panel could replace the clinical history. However, there are situations where this gene panel may be useful, such as confirmation of the history in clinical practice when deciding whether to start a patient on a therapy targeted against exacerbations, or in clinical trials to objectively confirm the frequent exacerbator phenotype.

### Strengths of the study

This study had several strengths. First, it included a derivative and a validating cohort of very well characterized patients followed prospectively using the same procedure for data gathering and analyses. Second, the simultaneous measurement of gene expression in two anatomical compartments (sputum and blood) provides information about the potential contribution of local versus systemic changes in the genesis of COPD exacerbations. Our data suggests that it is important to not only study the lungs, but also the systemic compartment when attempting to explain the pathogenesis of exacerbations. The differentially regulated genes reported here in the blood can be further investigated to understand the altered immunobiology in COPD frequent exacerbators.

### Limitations of the study

There are several potential limitations. First, the timing of sample collection in relation to exacerbations. We were extremely careful to collect samples from patients during the stable state, at least 4 weeks after an exacerbation, to avoid gene expression changes that were due to episodes of exacerbation themselves. The lack of induced sputum signals indicates that we were successful in this regard. Secondly, it could be argued that therapy, most notably inhaled corticosteroids, could have influenced the gene expression. More COPD subjects were taking inhaled corticosteroids in the frequent exacerbator phenotype (88.2% - see Table 1), likely because treatment guidelines suggest that these drugs should be prescribed to such patients. Whole blood gene expression in the intermediate group who had similar inhaled corticosteroid usage (80.3%) compared with the frequent exacerbators, but had a lower level of exacerbations, showed only 21 genes that were different compared to zero exacerbators, which is lower than the comparison of frequent exacerbators with zero exacerbators (150 genes). This demonstrates that any inhaled corticosteroid effect on gene expression was low and did not account for the major differences in gene expression. Thirdly, it could be argued that the study has little clinical applicability. However, improved understanding of pathways associated with frequent exacerbations may lead to the development of novel therapies targeting immune defects in this subset of patients. Our results provide insights into a number of pathways that provide the basis for future investigations.

It would be of interest to study the genes reported here longitudinally to observe changes over time. It is probable that gene expression patterns in COPD patients change over time, and this may be associated with a change in clinical phenotype such as frequency of exacerbations.

## Conclusions

We have demonstrated changes in the systemic immune function associated with the frequent exacerbator phenotype. There was down-regulation of lymphocyte function and a shift towards pro-apoptosis mechanisms in the peripheral blood of this phenotype. These may be important mechanisms that are responsible for the frequent exacerbation events observed in these patients and potentially their modulation could lead to a decrease in the number and/or duration of the episodes. More importantly, this study shows that the frequent exacerbator phenotype may have a biological underpinning and is not the product of simple perception or type of health delivery bias.

## Supporting Information

File S1 **Contains supporting information for methods and results sections, and Tables S1, S2, S3, S4 and S5.**
(DOCX)

## References

[1] Wedzicha JA, Seemungal TA (2007) COPD exacerbations: defining their cause and prevention. Lancet 370: 786–96. 17765528 10.1016/S0140-6736(07)61382-8 PMC7134993

[2] Papi A, Bellettato CM, Braccioni F, Romagnoli M, Casolari P, et al (2006) Infections and airway inflammation in chronic obstructive pulmonary disease severe exacerbations. Am J Respir Crit Care Med 173: 1114–21. 16484677 10.1164/rccm.200506-859OC

[3] Hurst JR, Vestbo J, Anzueto A, Locantore N, Müllerova H, et al (2010) Evaluation of COPD Longitudinally to Identify Predictive Surrogate Endpoints (ECLIPSE) Investigators. Susceptibility to exacerbation in chronic obstructive pulmonary disease. N Engl J Med 363: 1128–38. 20843247 10.1056/NEJMoa0909883

[4] Seemungal TA, Donaldson GC, Paul EA, Bestall JC, Jeffries DJ, et al (1998) Effect of exacerbation on quality of life in patients with chronic obstructive pulmonary disease. Am J Respir Crit Care Med 157: 1418–22. 9603117 10.1164/ajrccm.157.5.9709032

[5] Soler-Cataluña JJ, Martínez-Garcia MA, RománSánchez P, Salcedo E, Navarro M, et al (2005) Severe acute exacerbations and mortality in patients with chronic obstructive pulmonary disease. Thorax 60: 925–931. 16055622 10.1136/thx.2005.040527 PMC1747235

[6] Vestbo J, Anderson W, Coxson HO, Crim C, Dawber F, et al (2008) ECLIPSE investigators. Evaluation of COPD Longitudinally to Identify Predictive Surrogate End-points (ECLIPSE). Eur Respir J 31: 869–73. 18216052 10.1183/09031936.00111707

[7] Singh SD, Fox SM, Tal-Singer R, Plumb J, Bates S, et al (2011) Induced Sputum Genes Associated with Spirometric and Radiological Disease Severity in COPD ex-smokers. Thorax 65: 764–74. 10.1136/thx.2010.153767 21441172

[8] Simpson TR, Quezada SA, Allison JP (2010) Regulation of CD4 T cell activation and effector function by inducible costimulator (ICOS). Curr Opin Immunol 22: 326–32. 20116985 10.1016/j.coi.2010.01.001

[9] Sundberg EJ, Deng L, Mariuzza RA (2007) TCR recognition of peptide/MHC class II complexes and superantigens. SeminImmunol 19: 262–71. 10.1016/j.smim.2007.04.006 PMC2949352 17560120

[10] Macian F (2005) NFAT proteins: key regulators of T-cell development and function. Nat Rev Immunol 5: 472–84. 15928679 10.1038/nri1632

[11] Rygiel TP, Rijkers ES, de Ruiter T, Stolte EH, van der Valk M, et al (2009) Lack of CD200 enhances pathological T cell responses during influenza infection. J Immunol 183: 1990–6. 19587022 10.4049/jimmunol.0900252

[12] Saitou M, Sugimoto J, Hatakeyama T, Russo G, Isobe M, et al (2000) Identification of the TCL6 genes within the breakpoint cluster region on chromosome 14q32 in T-cell leukemia. Oncogene 19: 2796–802. 10851082 10.1038/sj.onc.1203604

[13] Ying H, Li Z, Yang L, Zhang J (2011) Syk mediates BCR- and CD40-signaling integration during B cell activation. Immunobiology 216: 566–70. 21074890 10.1016/j.imbio.2010.09.016 PMC3075491

[14] Hasler P, Zouali M (2001) B cell receptor signaling and autoimmunity. FASEB J 15: 2085–98. 11641235 10.1096/fj.00-0860rev

[15] Erdei A, Isaák A, Török K, Sándor N, Kremlitzka M, et al (2009) Expression and role of CR1 and CR2 on B and T lymphocytes under physiological and autoimmune conditions. MolImmunol 46: 2767–73. 10.1016/j.molimm.2009.05.181 19559484

[16] Kawasaki N, Rademacher C, Paulson JC (2011) CD22 regulates adaptive and innate immune responses of B cells. J Innate Immun 3: 411–9. 21178327 10.1159/000322375 PMC3130896

[17] Maurice MM, Lankester AC, Bezemer AC, Geertsma MF, Tak PP, et al (1997) Defective TCR-mediated signaling in synovial T cells in rheumatoid arthritis. J Immunol 159: 2973–8. 9300721

[18] Jury EC, Kabouridis PS, Abba A, Mageed RA, Isenberg DA (2003) Increased ubiquitination and reduced expression of LCK in T lymphocytes from patients with systemic lupus erythematosus. Arthritis Rheum 48: 1343–54. 12746907 10.1002/art.10978

[19] Grundy S, Plumb J, Lea S, Kaur M, Ray D, et al (2013) Down regulation of T cell receptor expression in COPD pulmonary CD8 cells. PLoS One 8: e71629. 23977094 10.1371/journal.pone.0071629 PMC3747211

[20] Hogg JC, Chu F, Utokaparch S, Woods R, Elliott WM, et al (2004) The nature of small-airway obstruction in chronic obstructive pulmonary disease. N Engl J Med 350: 2645–53. 15215480 10.1056/NEJMoa032158

[21] Zinkel S, Gross A, Yang E (2006) BCL2 family in DNA damage and cell cycle control. Cell Death Differ 13: 1351–9. 16763616 10.1038/sj.cdd.4401987

[22] Ware CF, VanArsdale S, VanArsdale TL (1996) Apoptosis mediated by the TNF-related cytokine and receptor families. J Cell Biochem 60: 47–55. 8825415 10.1002/(SICI)1097-4644(19960101)60:1%3C47::AID-JCB8%3E3.0.CO;2-3

[23] Demedts IK, Demoor T, Bracke KR, Joos GF, Brusselle GG, et al (2006) Role of apoptosis in the pathogenesis of COPD and pulmonary emphysema. Respir Res 7: 53. 16571143 10.1186/1465-9921-7-53 PMC1501017

[24] Marlow LA, von Roemeling CA, Cooper SJ, Zhang Y, Rohl SD, et al (2012) Foxo3a drives proliferation in anaplastic thyroid carcinoma through transcriptional regulation of cyclin A1: a paradigm shift that impacts current therapeutic strategies. J Cell Sci 125 (Pt 18): 4253–63. 10.1242/jcs.097428 PMC3516436 22718346

[25] Weckbach LT, Gola A, Winkelmann M, Jakob SM, Groesser L, et al (2014) The cytokine midkine supports neutrophil trafficking during acute inflammation by promoting adhesion via β2 integrins (CD11/CD18). Blood 123: 1887–96. 24458438 10.1182/blood-2013-06-510875

[26] Lee PL, Kohler JJ, Pfeffer SR (2009) Association of beta-1,3-N-acetylglucosaminyltransferase 1 and beta-1,4-galactosyltransferase 1, trans-Golgi enzymes involved in coupled poly-N-acetyllactosamine synthesis. Glycobiology 19: 655–64. 19261593 10.1093/glycob/cwp035 PMC2682609

[27] Ma C, Staudt LM (1996) LAF-4 encodes a lymphoid nuclear protein with transactivation potential that is homologous to AF-4, the gene fused to MLL in t(4;11) leukemias. Blood 87: 734–45. 8555498

[28] Chaya T, Shibata S, Tokuhara Y, Yamaguchi W, Matsumoto H, et al (2011) Identification of a negative regulatory region for the exchange activity and characterization of T332I mutant of Rho guanine nucleotide exchange factor 10 (ARHGEF10). J Biol Chem 286: 29511–20. 21719701 10.1074/jbc.M111.236810 PMC3190991

[29] Bhattacharya S, Tyagi S, Srisuma S, Demeo DL, Shapiro SD, et al (2011) Peripheral blood gene expression profiles in COPD subjects. J Clin Bioinforma 1: 12. 21884629 10.1186/2043-9113-1-12 PMC3164605

[30] Bahr TM, Hughes GJ, Armstrong M, Reisdorph R, Coldren CD, et al (2013) Peripheral blood mononuclear cell gene expression in chronic obstructive pulmonary disease. Am J Respir Cell Mol Biol 49: 316–23. 23590301 10.1165/rcmb.2012-0230OC PMC3824029

[31] Otsuki M, Fukami K, Kohno T, Yokota J, Takenawa T (1999) Identification and characterization of a new phospholipase C-like protein, PLC-L(2). Biochem Biophys Res Commun 266: 97–103. 10581172 10.1006/bbrc.1999.1784

