# Supplementary material for: Altered Gene Expression in Blood and Sputum in COPD Frequent Exacerbators in the ECLIPSE Cohort
Source: PLoS One. 2014 Sep 29;9(9):e107381. doi: 10.1371/journal.pone.0107381 (PMC4179270; doi:10.1371/journal.pone.0107381)
Supplement: File S1 — Contains supporting information for methods and results sections, and Tables S1, S2, S3, S4 and S5. (DOCX) [file pone.0107381.s001.docx]

**Supporting information**

**Methods**

**Sputum Induction And Processing**

Sputum induction was performed using 3% saline given as 3 nebulisations each lasting for 7 minutes. Selected sputum was mixed with 0.1% DTT on ice in a ratio of 4:1 and processed to obtain a cell pellet. The cell pellet was re-suspended in cold PBS so that a cell count could be performed and a cytopsin slide prepared for differential count. The suspension was then centrifuged at 4^o^C for 5 minutes (400-500 x g) to obtain a pellet that was suspended in a minimum of 1 ml cold TRIzol reagent (Invitrogen, Paisley, UK) to lyse the cells and stored -70^o^C. Cytospin preparations were air dried, fixed with methanol and stained with Rapi-diff (Triangle, Skelmersdale, UK). Five hundred leukocytes were counted by two independent readers at a central laboratory (at Dr Rennard’s lab in University of Nebraska, USA) and the results expressed as a percentage of the total leucocyte count, and a total cell number / ml.

**RNA Isolation from sputum**

Trizol lysates were thawed to room temperature. 1/5^th^ volume of chloroform was added before centrifugation at 14,000rpm for 3minutes. The aqueous phase was removed, an equal volume of 70% ethanol added and mixed. Lysate was applied to RNeasy mini-columns, processed according to manufacturers guidelines (including on-column DNase-I treatment) and RNA eluted in RNase-free water (Ambion). The quantity of extracted RNA was determined by spectrophotometry and quality assessed using an Agilent 2100 Bioanalyzer (Agilent, South Plainfield, NJ, USA).

**RNA Isolation from whole blood**

PAXGene tubes were gently inverted 8–10 times to ensure mixture of blood with the stabilization reagent, and then incubated for 2 hours at room temperature to ensure complete penetration of the PAXgene reagent into the blood cells, after which they were transferred initially to -20^o^C and then to -70^o^C for storage.

PAXGene tubes were thawed at room temperature overnight with gentle mixing, then centrifuged for 10mins at 4,000g, and supernatants discarded. The pellets were resuspended in 325µl of BR1 buffer (Qiagen) containing protein K, and mixed gently. Tubes were then loaded onto a BiRobot 8000 automation system (Qiagen) and run according to manufacturer’s guidelines. Following RNA elution, samples were heated to 80^o^C for 10mins and cooled on ice. The quantity of extracted RNA was determined by spectrophotometry and quality assessed using an Agilent 2100 Bioanalyzer (Agilent, South Plainfield, NJ, USA).

**Microarray Analysis**

RNA samples were adjusted to 10ng/ul, and 50ng of total RNA was labelled using the Nugen Ovation RNA Amplification v2 procedure following manufacturer’s instructions. 3.75ug of biotin labelled cDNA was hybridised to HG_Plus_2.0 GeneChips (Affymetrix, Santa Clara, CA, USA) following standard procedures. Arrays were scanned using a GeneChip Scanner 3000 and fluorescent intensity for each feature on the array was obtained by using GeneChip Operating Software (Affymetrix, Santa Clara, CA, USA).

Standard MAS5.0 Affymetrix quality control criteria were examined to determine the quality of the GeneChip data. A gender check was performed as an additional quality control measure and to ensure that samples had been correctly annotated by accessing expression levels of DDX3Y (205000_at) which is male specific and XIST (224589_at) which is female specific.

Standard MAS5.0 Affymetrix quality control criteria were examined to determine the quality of the GeneChip data. The raw signal intensities (Cel files) for each scan were imported into gene expression analysis software, Resolver version 4.0 (Rosetta Biosoftware, Seattle, USA). Signal extraction was performed with Resolver and the normalized data were then exported for further analysis. Principal Component Analysis (PCA) was performed on detected probesets (as defined in Rosetta Resolver) using SIMCA-P (Umetrics). Mixed model ANOVA analysis was carried out using SAS analysis software. The gene expression values were log-transformed using LOG_10_(x+1)and filtering was applied to remove probe-sets that showed only nominal expression; for each probe-set the median value was calculated, and probe sets with intensity values < 30 were then removed.

**Real time PCR**

RNA samples were diluted to make stock solutions of 5ng/ul. 25ng of total RNA was amplified using the NuGEN Ovation RNA Amplification System v2. 10ng of amplified was then used for RT-PCR analysis in 10ul reactions. Triplicate reactions were performed for each primer/probe set, while no RNA controls were included to monitor genomic DNA contamination. Each reaction included 900nM of probe, 100nM of each primer and Universal Master Mix-no AmpErase UNG (Applied Biosystems). Primers and probes were designed using Primer 2.1 software (ABI). Results were generated using the 5’ nuclease assay (TaqMan) using the ABI 7900HT Sequence Detection System (Applied Biosystems, Foster City, CA) with 2 housekeeping genes (Cyclophilin A and GAPDH).The geometric mean of calculated abundance levels were calculated for technical replicates thus giving one abundance measure per sample.

**Supplementary information regarding Fig 2 in main text**

Of 21 genes differentially expressed in intermediate group compared to zero exacerbators, 4 were also differentially expressed in the frequent exacerbators compared to zero exacerbators: FAM3C, FCRL2, KLF12, N4BP2.

**Table S1:**

Demographics of the COPD subjects who provided samples for microarray analysis at the start of the study (n=138). Blood counts are mean values (X10^9^cells / L).

| Number | 138 |
| --- | --- |
| Stage II (%) | 50.0 |
| Stage III (%) | 41.3 |
| Stage IV (%) | 8.7 |
| Male (%) | 66.7 |
| Mean Age | 65.0 |
| Mean Pack Years | 48.4 |
| Percent Predicted FEV1 (%) | 49.9 |
| LABA (%) | 79.7 |
| Steroid (%) | 76.1 |
| Blood WBC | 7.45 |
| Blood neutrophil count | 4.90 |
| Blood eosinophil count | 0.23 |
| Blood leukocyte count | 1.82 |
| Blood Monocyte count | 0.47 |

**Table S2**

Differentially expressed gene sets in frequent compared to zero exacerbators with a fold change +/- 1.5 and p<0.01. Positive fold change = increase in frequent exacerbators compared to zero exacerbators. Negative fold change = decrease in frequent exacerbators compared to zero exacerbators. E-03 denotes (X10^-3^)

| Gene Name | Affy. ID | Fold change | p value | Tissue |
| --- | --- | --- | --- | --- |
| LOC284723 | 232245_at | 1.71 | 8.82E-03 | SPUTUM |
| ANKRD28 | 213035_at | 1.71 | 7.61E-03 | SPUTUM |
| LOC284723 | 1559977_a_at | 1.65 | 8.62E-03 | SPUTUM |
| LDHAL6B | 210712_at | 1.55 | 1.32E-03 | SPUTUM |
| MIA | 206560_s_at | 1.53 | 8.44E-03 | SPUTUM |
| RPS16 | 226130_at | 1.48 | 5.29E-03 | SPUTUM |
| CLEC4D | 1552773_at | 1.46 | 7.92E-03 | SPUTUM |
| SYNPO | 202796_at | -1.48 | 7.21E-04 | SPUTUM |
| PAX3 | 207680_x_at | -1.49 | 8.68E-03 | SPUTUM |
| OASL | 205660_at | -1.54 | 9.25E-03 | SPUTUM |
| RHCE | 215819_s_at | 2.25 | 9.79E-03 | BLOOD |
| CCNA1 | 205899_at | 2.23 | 8.58E-04 | BLOOD |
| ITGB2 | 236988_x_at | 2.21 | 7.93E-03 | BLOOD |
| ADORA3 | 223660_at | 2.03 | 1.94E-03 | BLOOD |
| CCL23 | 210548_at | 1.97 | 8.37E-03 | BLOOD |
| AP3S2 | 228791_at | 1.79 | 2.17E-03 | BLOOD |
| CGI-14 | 219082_at | 1.71 | 8.94E-04 | BLOOD |
| FBXL13 | 1553798_a_at | 1.66 | 2.11E-03 | BLOOD |
| IGSF2 | 244652_at | 1.66 | 6.29E-04 | BLOOD |
| PLEKHA2 | 238013_at | 1.61 | 6.05E-03 | BLOOD |
| MGC31963 | 1558693_s_at | 1.61 | 4.63E-04 | BLOOD |
|  | 235596_at | 1.59 | 4.57E-03 | BLOOD |
| ADAM15 | 1555896_a_at | 1.59 | 5.35E-03 | BLOOD |
|  | 235126_at | 1.59 | 3.74E-03 | BLOOD |
| IGSF2 | 207167_at | 1.59 | 2.12E-03 | BLOOD |
| CST4 | 206994_at | 1.58 | 1.46E-03 | BLOOD |
| 13CDNA73 | 214319_at | 1.58 | 6.92E-03 | BLOOD |
| TNNI2 | 206393_at | 1.57 | 1.55E-03 | BLOOD |
| AKIP | 222677_x_at | 1.56 | 3.93E-03 | BLOOD |
| RAPGEF2 | 238176_at | 1.56 | 7.67E-03 | BLOOD |
| AP3S2 | 217548_at | 1.54 | 1.13E-03 | BLOOD |
| CATSPER1 | 1552335_at | 1.54 | 5.25E-04 | BLOOD |
| EPAS1 | 242868_at | 1.53 | 9.39E-04 | BLOOD |
| CHST13 | 239647_at | 1.53 | 5.80E-03 | BLOOD |
| C9orf39 | 241696_at | 1.52 | 1.07E-03 | BLOOD |
| ENTPD2 | 207372_s_at | 1.51 | 4.39E-04 | BLOOD |
| CDT1 | 209832_s_at | 1.50 | 5.30E-03 | BLOOD |
| FLJ30473 | 244084_at | 1.50 | 1.21E-03 | BLOOD |
|  | 228919_at | 1.50 | 9.11E-03 | BLOOD |
| TCF4 | 213891_s_at | -1.50 | 1.03E-03 | BLOOD |
| ICOS | 210439_at | -1.50 | 1.55E-03 | BLOOD |
| TPD52 | 201689_s_at | -1.50 | 2.63E-03 | BLOOD |
| SESN1 | 218346_s_at | -1.51 | 3.96E-04 | BLOOD |
| CCDC2 | 219174_at | -1.51 | 3.47E-03 | BLOOD |
| CD72 | 215925_s_at | -1.51 | 4.51E-03 | BLOOD |
| COBLL1 | 229598_at | -1.51 | 5.92E-03 | BLOOD |
|  | 1562412_at | -1.52 | 6.84E-04 | BLOOD |
| KIAA1914 | 226829_at | -1.52 | 2.64E-03 | BLOOD |
| ZNF83 | 221645_s_at | -1.52 | 5.51E-03 | BLOOD |
| SMC2L1 | 204240_s_at | -1.52 | 5.73E-03 | BLOOD |
| TTC12 | 215598_at | -1.52 | 5.46E-03 | BLOOD |
| RALGPS2 | 232112_at | -1.52 | 6.58E-03 | BLOOD |
| TIP120B | 213547_at | -1.52 | 3.85E-03 | BLOOD |
| ZNF507 | 235618_at | -1.52 | 1.25E-04 | BLOOD |
| SLC9A7 | 1558105_a_at | -1.52 | 2.83E-03 | BLOOD |
|  | 230152_at | -1.53 | 2.98E-03 | BLOOD |
| RAB30 | 228003_at | -1.53 | 1.09E-03 | BLOOD |
| FLJ22531 | 238593_at | -1.53 | 3.76E-03 | BLOOD |
| STRBP | 229513_at | -1.53 | 2.18E-05 | BLOOD |
| OXCT1 | 202780_at | -1.53 | 2.48E-03 | BLOOD |
| TTC9 | 213172_at | -1.53 | 2.02E-03 | BLOOD |
|  | 237627_at | -1.53 | 2.93E-04 | BLOOD |
| FCRL3 | 241278_at | -1.53 | 8.25E-03 | BLOOD |
| BACH2 | 236307_at | -1.53 | 2.59E-03 | BLOOD |
| FLJ22814 | 220674_at | -1.53 | 7.62E-03 | BLOOD |
| TNFRSF13C | 243780_at | -1.53 | 6.81E-04 | BLOOD |
| KLF12 | 227261_at | -1.54 | 2.67E-03 | BLOOD |
| LARGE | 215543_s_at | -1.54 | 9.66E-03 | BLOOD |
| Cep290 | 221683_s_at | -1.54 | 3.63E-03 | BLOOD |
| LAF4 | 232286_at | -1.54 | 2.53E-03 | BLOOD |
| CHML | 226350_at | -1.54 | 5.07E-03 | BLOOD |
| C3orf6 | 228693_at | -1.55 | 2.72E-03 | BLOOD |
| B3GNT6 | 203188_at | -1.55 | 7.01E-05 | BLOOD |
| TPD52 | 236655_at | -1.55 | 2.52E-03 | BLOOD |
| TRBV16 | 217060_at | -1.55 | 1.22E-03 | BLOOD |
| DPF3 | 238532_at | -1.55 | 4.03E-04 | BLOOD |
| TAF4B | 235020_at | -1.55 | 2.15E-04 | BLOOD |
| FAM3C | 201889_at | -1.55 | 2.66E-03 | BLOOD |
| C3orf4 | 239146_at | -1.55 | 4.67E-03 | BLOOD |
| ST8SIA1 | 210073_at | -1.55 | 7.00E-03 | BLOOD |
| SPATA5 | 229075_at | -1.55 | 4.70E-04 | BLOOD |
| CACNB2 | 1559419_at | -1.55 | 3.37E-03 | BLOOD |
| THNSL1 | 222931_s_at | -1.55 | 3.59E-03 | BLOOD |
| LOC56902 | 242905_at | -1.55 | 4.06E-04 | BLOOD |
| ZNF600 | 242463_x_at | -1.56 | 3.57E-03 | BLOOD |
| HLCS | 1562399_at | -1.56 | 9.70E-03 | BLOOD |
| BLK | 206255_at | -1.56 | 3.09E-03 | BLOOD |
| IGHV4-4 | 241074_at | -1.57 | 5.11E-04 | BLOOD |
| LAF4 | 244696_at | -1.58 | 1.95E-03 | BLOOD |
| HTPAP | 226384_at | -1.58 | 5.14E-05 | BLOOD |
| EBF | 232204_at | -1.58 | 6.09E-03 | BLOOD |
| ZNF177 | 207417_s_at | -1.58 | 6.73E-03 | BLOOD |
| KS | 219133_at | -1.59 | 4.48E-03 | BLOOD |
| CD22 | 38521_at | -1.59 | 1.24E-03 | BLOOD |
| RPL5 | 216044_x_at | -1.59 | 4.70E-04 | BLOOD |
| VprBP | 226477_at | -1.59 | 2.07E-03 | BLOOD |
| ZXDA | 243521_at | -1.59 | 8.28E-03 | BLOOD |
| N4BP2 | 230248_x_at | -1.59 | 2.05E-03 | BLOOD |
|  | 234428_at | -1.59 | 4.32E-03 | BLOOD |
| FREB | 235401_s_at | -1.61 | 3.66E-03 | BLOOD |
| RPL30 | 228047_at | -1.61 | 9.60E-03 | BLOOD |
| SLC9A7 | 226550_at | -1.61 | 3.09E-04 | BLOOD |
| EBF | 233261_at | -1.61 | 8.20E-03 | BLOOD |
| LOC283663 | 230245_s_at | -1.62 | 5.70E-04 | BLOOD |
| COBLL1 | 203642_s_at | -1.62 | 2.56E-03 | BLOOD |
| CD22 | 204581_at | -1.62 | 1.12E-03 | BLOOD |
| GPM6A | 236024_at | -1.62 | 6.22E-03 | BLOOD |
| BRDG1 | 220059_at | -1.62 | 3.56E-04 | BLOOD |
| ABCB4 | 207819_s_at | -1.62 | 1.41E-03 | BLOOD |
| C17orf25 | 237389_at | -1.62 | 1.31E-03 | BLOOD |
| FLJ20097 | 1554216_at | -1.62 | 4.06E-04 | BLOOD |
| TBC1D4 | 203387_s_at | -1.62 | 8.23E-03 | BLOOD |
| LOC283663 | 230648_at | -1.63 | 1.02E-03 | BLOOD |
| INADL | 223681_s_at | -1.63 | 2.50E-03 | BLOOD |
|  | 241505_at | -1.63 | 5.99E-03 | BLOOD |
| MS4A1 | 228599_at | -1.64 | 3.19E-03 | BLOOD |
| C20orf17 | 220213_at | -1.64 | 1.44E-03 | BLOOD |
|  | 1558185_at | -1.64 | 8.20E-04 | BLOOD |
|  | 1564160_at | -1.64 | 5.49E-03 | BLOOD |
|  | 243810_at | -1.64 | 1.62E-05 | BLOOD |
| KCNQ5 | 242976_at | -1.65 | 2.01E-03 | BLOOD |
| FREB | 235372_at | -1.66 | 6.19E-04 | BLOOD |
| LOC284749 | 236854_at | -1.66 | 5.27E-03 | BLOOD |
| LAF4 | 227198_at | -1.66 | 1.48E-04 | BLOOD |
| FVT1 | 229850_at | -1.67 | 3.52E-03 | BLOOD |
| LAMC1 | 243591_at | -1.67 | 3.19E-03 | BLOOD |
| BANK1 | 1558662_s_at | -1.67 | 2.49E-04 | BLOOD |
| CD19 | 206398_s_at | -1.67 | 2.04E-03 | BLOOD |
| BDP1 | 226290_at | -1.67 | 9.95E-03 | BLOOD |
| CELSR1 | 41660_at | -1.68 | 4.96E-03 | BLOOD |
| EBF | 227646_at | -1.68 | 2.23E-03 | BLOOD |
| PCDH9 | 219737_s_at | -1.69 | 5.92E-04 | BLOOD |
| IGHG1 | 228518_at | -1.71 | 7.54E-03 | BLOOD |
| IGHG3 | 230877_at | -1.71 | 7.63E-03 | BLOOD |
| RAB30 | 228390_at | -1.71 | 9.86E-05 | BLOOD |
| ZNF677 | 228974_at | -1.72 | 2.25E-03 | BLOOD |
| CRYBB2 | 206777_s_at | -1.73 | 2.03E-03 | BLOOD |
| LAF4 | 1566880_at | -1.73 | 7.04E-04 | BLOOD |
| RAB30 | 229072_at | -1.73 | 6.82E-04 | BLOOD |
| ARMC8 | 219094_at | -1.74 | 1.21E-03 | BLOOD |
| LAF4 | 241577_at | -1.74 | 5.74E-04 | BLOOD |
| BLK | 244394_at | -1.74 | 3.10E-04 | BLOOD |
| OSBPL10 | 219073_s_at | -1.75 | 2.39E-03 | BLOOD |
| PMS2L4 | 215192_at | -1.75 | 1.13E-04 | BLOOD |
| AGPAT5 | 232007_at | -1.77 | 4.13E-03 | BLOOD |
| E2F5 | 221586_s_at | -1.79 | 1.49E-04 | BLOOD |
| CR2 | 205544_s_at | -1.79 | 3.69E-04 | BLOOD |
| FCRL1 | 243968_x_at | -1.79 | 7.91E-05 | BLOOD |
| IGHG3 | 222285_at | -1.79 | 1.35E-03 | BLOOD |
| RALGPS2 | 227224_at | -1.80 | 1.58E-03 | BLOOD |
| MS4A1 | 228592_at | -1.80 | 3.44E-05 | BLOOD |
| SDK2 | 242064_at | -1.81 | 6.65E-04 | BLOOD |
| BRDG1 | 1554343_a_at | -1.81 | 2.00E-04 | BLOOD |
| BANK1 | 219667_s_at | -1.83 | 9.45E-05 | BLOOD |
|  | 236103_at | -1.83 | 9.91E-03 | BLOOD |
| FCRL5 | 224406_s_at | -1.83 | 3.06E-03 | BLOOD |
| KIAA0125 | 206478_at | -1.84 | 2.66E-04 | BLOOD |
| MGC40069 | 243602_at | -1.84 | 3.86E-03 | BLOOD |
| EBF | 244876_at | -1.85 | 1.81E-03 | BLOOD |
|  | 1568807_a_at | -1.85 | 4.53E-05 | BLOOD |
|  | 1562453_at | -1.86 | 3.85E-03 | BLOOD |
| PLEKHG1 | 226122_at | -1.89 | 2.69E-03 | BLOOD |
| PPAT | 209433_s_at | -1.90 | 9.19E-04 | BLOOD |
| FCRL2 | 1563674_at | -1.91 | 1.09E-04 | BLOOD |
| OSBPL10 | 239292_at | -1.96 | 3.26E-04 | BLOOD |
| KLHL14 | 228377_at | -2.00 | 1.35E-03 | BLOOD |
| ABCB4 | 1570505_at | -2.04 | 1.79E-03 | BLOOD |
| COL4A3 | 222073_at | -2.05 | 1.19E-03 | BLOOD |
| FCRL1 | 235982_at | -2.06 | 5.76E-04 | BLOOD |
| FCRL2 | 221239_s_at | -2.06 | 5.34E-04 | BLOOD |
| CD200 | 209583_s_at | -2.09 | 1.98E-03 | BLOOD |
|  | 230896_at | -2.20 | 5.72E-05 | BLOOD |
| HLA-DQB1 | 212999_x_at | -2.22 | 8.47E-03 | BLOOD |
|  | 239244_at | -2.27 | 3.13E-04 | BLOOD |
| TCL6 | 219840_s_at | -2.59 | 2.93E-04 | BLOOD |
| HLA-DQA1 | 236203_at | -3.27 | 3.19E-03 | BLOOD |

**Table S3**

Differentially expressed genes in intermediate compared to zero exacerbators with a fold change +/- 1.5 and p<0.01. Positive fold change = increase in intermediate group compared to zero exacerbators. Negative fold change = decrease in intermediate group compared to zero exacerbators. E-03 denotes (X10^-3^)

**Table S4**

Differentially expressed genes in frequent exacerbators compared to the intermediate group with a fold change +/- 1.5 and p<0.01. Positive fold change = increase in frequent exacerbators compared to intermediate group. Negative fold change = decrease in frequent exacerbators compared to intermediate group. E-03 denotes (X10^-3^)

**Table S5**

Genego analysis: List of significant pathways using FDR (false discovery rate) <0.05. E-04 denotes (x10^-4^ ). The number of genes found to be regulated within a pathway are stated.
